# Interferon Alpha Induces Establishment of Alphaherpesvirus Latency in Sensory Neurons *In Vitro*

**DOI:** 10.1371/journal.pone.0013076

**Published:** 2010-09-29

**Authors:** Nick De Regge, Nina Van Opdenbosch, Hans J. Nauwynck, Stacey Efstathiou, Herman W. Favoreel

**Affiliations:** 1 Department of Virology, Parasitology, and Immunology, Faculty of Veterinary Medicine, Ghent University, Merelbeke, Belgium; 2 Division of Virology, Department of Pathology, University of Cambridge, Cambridge, United Kingdom; Nanyang Technological University, Singapore

## Abstract

**Background:**

Several alphaherpesviruses, including herpes simplex virus 1 (HSV-1) and pseudorabies virus (PRV), establish lifelong latency in neurons of the trigeminal ganglion (TG). Although it is thought that efficient establishment of alphaherpesvirus latency is based on a subtle interplay between virus, neurons and the immune system, it is not clear which immune components are of major importance for the establishment of latency.

**Methodology/Principal Findings:**

Here, using an *in vitro* model that enables a natural route of infection, we show that interferon alpha (IFNalpha) has the previously uncharacterized capacity to induce a quiescent HSV-1 and PRV infection in porcine TG neurons that shows strong similarity to in vivo latency. IFNalpha induced a stably suppressed HSV-1 and PRV infection in TG neurons *in vitro*. Subsequent treatment of neurons containing stably suppressed virus with forskolin resulted in reactivation of both viruses. HSV and PRV latency *in vivo* is often accompanied by the expression of latency associated transcripts (LATs). Infection of TG neurons with an HSV-1 mutant expressing LacZ under control of the LAT promoter showed activation of the LAT promoter and RT-PCR analysis confirmed that both HSV-1 and PRV express LATs during latency *in vitro*.

**Conclusions/Significance:**

These data represent a unique *in vitro* model of alphaherpesvirus latency and indicate that IFNalpha may be a driving force in promoting efficient latency establishment.

## Introduction

Alphaherpesviruses are a subfamily of the herpesviruses containing closely related human and animal pathogens, including human HSV-1 (cold sores, corneal blindness, and encephalitis) and important animal viruses such as the porcine pseudorabies virus (PRV) and bovine herpesvirus 1 (BoHV-1; respiratory symptoms, abortions, and/or neurological symptoms).

Cycles of latency and reactivation arguably constitute the most important and fascinating hallmarks of alphaherpesvirus infections. Alphaherpesviruses generally establish latency in sensory neurons, and neurons of the trigeminal ganglion (TG) are the predominant site of latency for several important alphaherpesviruses, such as HSV-1, PRV, and BoHV-1 [1]–[3]. Although there is direct and indirect evidence to support the general concept that alphaherpesvirus latency and reactivation is based on a subtle interplay between virus, neurons and the immune system, many questions remain about the immune components that are involved in the establishment of latency [4].

It is becoming increasingly clear that the innate immune system has an important role in controlling alphaherpesvirus infections. Type I interferons (IFNalpha and -beta) are among the first immune effectors produced upon alphaherpesvirus infection [5], [6] and it has been shown that they are important in limiting viral replication and spread in vitro, but also in vivo at the periphery during initial infection and during reactivation [7]–[9]. Furthermore, type I interferons have been shown to be present at the periphery [7] and within the ganglion [10] around the time point that latency is established.

In the current study, using an *in vitro* two-chamber model that enables a natural route of alphaherpesvirus infection of porcine TG neurons [11], [12], we report that treatment of TG neurons with IFNalpha is sufficient to induce a quiescent HSV-1 and PRV infection *in vitro* that shows strong similarities to in vivo latency, thereby providing a novel and unique in vitro model to study HSV/PRV latency and reactivation and suggesting that IFNalpha may represent a key immune component involved in efficient establishment of alphaherpesvirus latency in sensory neurons.

## Materials and Methods

### Ethics statement

Trigeminal ganglia were derived from animals that were euthanized at the Faculty of Veterinary Medicine, Ghent University, Belgium, according to FELASA guidelines (Federation of European Laboratory Animal Science Associations).

### Cells and viruses

Wild type PRV strain Becker [13] was propagated on Swine Testicle cells. Wild type HSV-1 strain F [14] and HSV-1 mutants SΔUS5-LacZ [15] and LbetaA [16] were propagated on Vero cells.

### Cultivation and inoculation of primary trigeminal ganglion neuronal cultures in a two-chamber model

Porcine trigeminal ganglia were excised from 2 to 4 week old piglets and dissociated by enzymatic digestion with 0.2% collagenase A (Roche)[17]. The harvested cells were resuspended in culture medium (MEM supplemented with 10% fetal bovine serum, 100 U/ml penicillin, 0.1 mg/ml streptomycin, 0.1 mg/ml kanamycin and 30 ng/ml nerve growth factor (Sigma)) and seeded in the inner chamber of an *in vitro* two-chamber model. The two-chamber model consists of a polystyrene cloning cylinder (Sigma) that is fixed with silicon grease on a collagen coated cover glass inserted in a 6 well plate [11]. The inside of the cylinder forms the inner chamber, the outside forms the outer chamber. One day after seeding, cultures are washed with RPMI (Gibco) to remove non-adherent cells and from then on, culture medium is changed three times a week. After two to three weeks of cultivation, when clear axon growth can be observed in the outer chamber, two-chamber models are ready for inoculation with virus. Inoculation with all viruses used was done by adding 2×10^7^ PFU to the outer chamber. For PRV, two hours after inoculation of the outer chamber, medium containing PRV was removed and the outer chamber was washed twice with culture medium. Afterwards, new culture medium supplemented with polyclonal antibodies to PRV and guinea pig complement (Sigma) was added to prevent continuous infection pressure from the outer chamber to neurons in the inner chamber. For HSV-1, the virus was removed at 48 h after inoculation by washing and new culture medium supplemented with monoclonal antibodies to HSV-1 gD and guinea pig complement was added.

### Antibodies, cytokines and chemicals

Polyclonal porcine FITC-labeled anti-PRV antibodies [18] were used to detect late PRV proteins gB, gD and gE [17]. Monoclonal mouse-anti-PRV gD antibody 13D12 was described earlier [18]. Polyclonal rabbit-anti-PRV IE180 antibody was a kind gift from E. Tabarés (Universidad Autonoma de Madrid, Spain). Monoclonal mouse-anti-HSV-1 gD (124/468) and –anti-HSV-1 ICP4 (sc56986) were purchased from Santa Cruz Biotechnology and the neuronal marker rabbit-anti-neurofilament 200 from Sigma. Texas red-labeled goat-anti-rabbit antibodies and FITC-labeled goat-anti-mouse antibodies were from Invitrogen. Recombinant porcine IFNalpha and IFNgamma were purchased from R&D and X-gal and forskolin were obtained from Sigma.

### Quantification of the percentage viral antigen or beta-galactosidase positive infected neurons

The ratio between viral antigen positive or beta-galactosidase positive neurons in the inner chamber to the number of axons in the outer chamber after different treatments was determined by calculating viral antigen positive neuronal cell bodies (immunofluorescence) or beta-galactosidase positive neurons (X-gal) in the inner chamber and immunofluorescently labeled axons in the outer chamber. For each experiment, at least 25 neurons with outgrowth to the outer chamber were examined. Data shown represent means ± s.e.m. of independent triplicate assays.

### Immunofluorescence staining procedures

Two-chamber systems to be used for immunofluorescent detection of viral antigen positive neurons were washed in PBS and fixed in 100% methanol for 20 min at −20°C. In two-chamber systems to be used for analysis of beta-galactosidase positive neurons in the inner chamber, the outer chamber was fixed in 4% paraformaldehyde in PBS for 10 min and subsequently permeabilized in 0.2% TritonX-100 in PBS for 2 min. All antibodies were diluted in PBS, all to a dilution of 1∶100. Cells were incubated with each antibody for 1 h at 37°C and were washed two times between all incubation steps and after the last incubation step.

### Detection of beta-galactosidase activity

Cells in the inner chamber of two-chamber systems were fixed in 2% paraformaldehyde-0.2% glutaraldehyde in PBS for 15 min at RT and subsequently incubated with staining buffer (0.01% Na-deoxycholate, 0.02% NP40, 2 mM MgCl_2_, 4.5 mM potassium ferricyanide, 4.5 mM potassium ferrocyanide in PBS) for 5 min at RT, followed by incubation with X-gal buffer (staining buffer supplemented with 1 mg/ml X-gal) for 4 h at 37°C. Afterwards, the inner chamber was washed twice with PBS and immediately analyzed by light microscopy.

### RT-PCR

RNA from cells grown in the inner chamber of two-chamber systems was isolated and purified using the Trizol Plus RNA purification kit (Invitrogen) according to manufacturer's instructions, followed by a DNaseI digestion to degrade any contaminating DNA. RNA was reverse transcribed using SuperScript III RT enzyme (Invitrogen) according to manufacturer's instructions. OligodT primers were used for reverse transcription of actin, PRV IE180, PRV gB, PRV LAT, HSV-1 ICP0, HSV-1 gB and HSV-1 gD RNA and a gene specific primer (GSP-LAT) that allows amplification of the 2 kb LAT intron (Table 1) was used for reverse transcription of HSV-1 LAT RNA. The cDNA was then amplified by PCR using the AccuPrime Taq DNA polymerase system (Invitrogen). The sequences of primers, annealing temperatures and predicted lengths of amplified fragments can be found in Table 1. Amplified PCR fragments were analyzed by agarose gel electrophoresis and staining with Ethidium Bromide. The specificity of amplified fragments was verified by predicted sizes and by sequencing fragments that were purified from the agarose gel. Purified fragments were subjected to cycle sequencing with a Big Dye Terminator Cycle Sequencing kit v1.1 (Applied Biosystems) and cycle sequencing reaction products were purified using ethanol precipitation and separated on an ABI Genetic Analyzer 310 (Applied Biosystems). Obtained fragment sequences were compared with the NCBI nucleotide collection (nr/nt) database using MegaBlast.

**Table 1:** RT-PCR specifications.

	primer		sequence (5′-3′)	annealing temp.	predicted length
porcine	actin	forward:	ATGCAGAAGGAGATCACGGC	50	199
		reverse:	AGTCCGCCTAGAAGCATTTG		
PRV	IE180	forward:	ACGCGAGAGGAAGTAGGGAG	57	393
		reverse:	GTACCTGCACCGCAGTGAAG		
	gB	forward:	CCTCCTCGACGATGCAGTTG	59	281
		reverse:	CACGAACCGCTTCACAGACC		
	LAT	forward:	CATAAAGCCAGTTGAAGACGGGG	59	526
		reverse:	TAGAGGGTCTTGGGGATGTTGG		
HSV-1	ICP0	forward:	GCCCACTATCAGGTACAC	55	301
		reverse:	CACGGAACTGTTCGAGAC		
	gB	forward:	TGGCGTCGGAAGAGAATCGG	59	213
		reverse:	AGCAGGTCGACGGCTTCTAC		
	gD	forward:	AGCCAAGGGCTCCTGTAAG	58	352
		reverse:	GTCCTGGATCGACGGTATGTG		
	GSP-LAT		TGGTGGACCAGACGGGAAAC		
	LAT	forward:	CCGCGATACATCCAACAC	53	383
		reverse:	GAACAGCCTCTGGATGAC		

Primer sequences and annealing temperatures (°C) used in RT-PCR and predicted length (bp) of amplified fragments.

### Confocal microscopy

Samples were analyzed on a Leica TCS SP2 laser scanning spectrum confocal system (Leica Microsystems GmbH) linked to a Leica DM IRBE microscope. Images were taken using a 63x oil objective (NA 1.40-0.60) at RT and using Leica confocal acquisition software. Adjustments of brightness and contrast were applied to the entire images and were done using Adobe Photoshop (Adobe Systems Inc.).

## Results

### PRV and HSV-1 proceed to productive replication in porcine TG neurons

**Figure 1:**
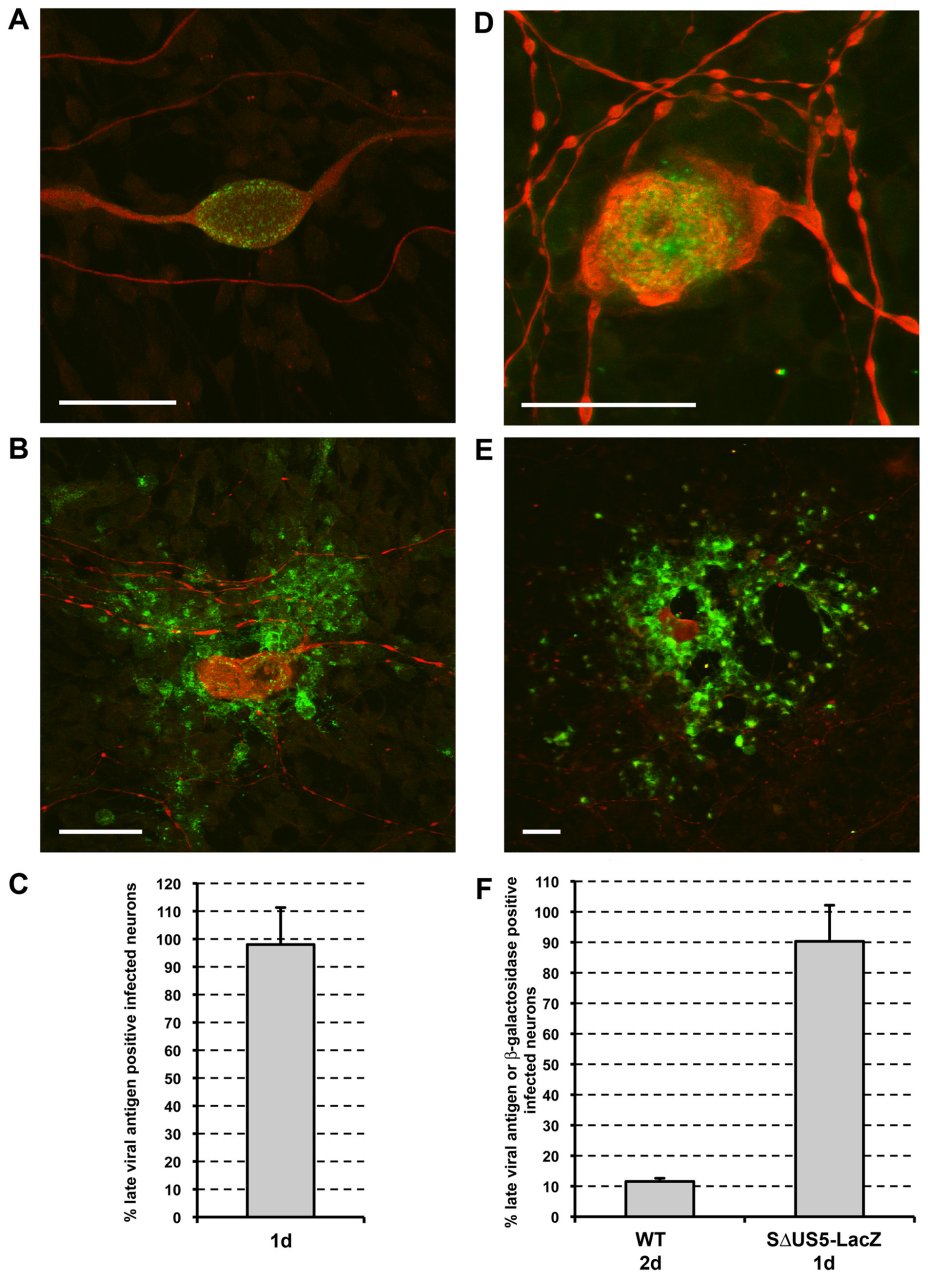
Productive replication of PRV and HSV-1 in porcine TG neurons. Confocal images of TG neuronal cultures in the inner chamber at 24hpi with PRV (A,B) and 48hpi with HSV-1 (D,E) stained for neurofilament (red) and late viral antigens (green) (bar  = 50 µm). Percentage of neurons with axons growing out to the outer chamber that show viral antigens at 24hpi with PRV (C) and 48hpi with wt HSV-1 (F, left bar) and beta-galactosidase activity at 24hpi with SΔUS5-LacZ HSV-1 (F, right bar). Data represent the mean ± s.e.m. of three independent experiments.

Porcine TG neurons grown in two-chamber models were infected with PRV or HSV-1 by addition of virus to the outer chamber. For PRV, at 24hpi, the vast majority of neurons (98%) that had axons growing in the outer chamber were positive for late viral antigens (Fig. 1C), indicating that PRV proceeds to productive infection in virtually all infected neurons. In some infected neurons at 24hpi, late PRV protein expression was limited to the neuronal cell body (Fig. 1A) while in others, infection had already spread to non-neuronal cells surrounding the cell body (Fig. 1B). Initiation of productive infection was less efficient when TG neurons were infected with HSV-1, but still resulted in 12% of the neurons with axonal outgrowth in the outer chamber being positive for the late HSV-1 antigen gD at 48hpi (Fig. 1F). As for PRV, some neurons showed HSV-1 gD expression limited to the neuronal cell body (Fig. 1D) while in others infection had already spread from the cell body to surrounding non-neuronal cells at this time point (Fig. 1E). To ensure that the substantial difference in percentage of productively infected neurons between HSV-1 and PRV was not due to the difference in antibodies used (polyclonal mixture for PRV versus monoclonal gD-specific for HSV-1), experiments with PRV were repeated using a PRV gD-specific monoclonal antibody. Again, the vast majority (75.3±6.2%) of neurons with axonal outgrowth in the outer chamber were positive at 24hpi with PRV. To analyze whether the limited percentage of productively HSV-1 infected neurons is due to a hampered HSV-1 entry in porcine TG neurons, neurons were infected with the SΔUS5-LacZ HSV-1 mutant carrying the reporter gene LacZ under control of the human CMV MIEP promoter inserted in the non-essential US5 gene [15]. X-gal staining of infected cultures showed that over 90% of neurons with axons growing into the outer chamber were beta-galactosidase positive at 24hpi (Fig. 1F), indicating that HSV-1 efficiently enters porcine TG neurons but does not efficiently initiate productive replication. To evaluate whether the block in productive HSV-1 replication occurs before or after expression of the earliest viral proteins (immediate early or IE proteins), HSV-1 experiments were repeated using an ICP4-specific monoclonal antibody. This resulted in a percentage of positive neurons (11.7±1.5%) similar to the percentage observed using gD-specific antibodies, indicating that the neurons that do not proceed to productive HSV-1 replication are halted before detectable ICP4 protein expression. Overall, these results indicate that upon viral entry, PRV efficiently proceeds to productive replication. For HSV-1, ±12% of infected neurons proceed to productive replication whereas the others are halted at a stage very early in infection.

### Interferon alpha suppresses PRV and HSV-1 productive replication in TG neurons for several days

**Figure 2:**
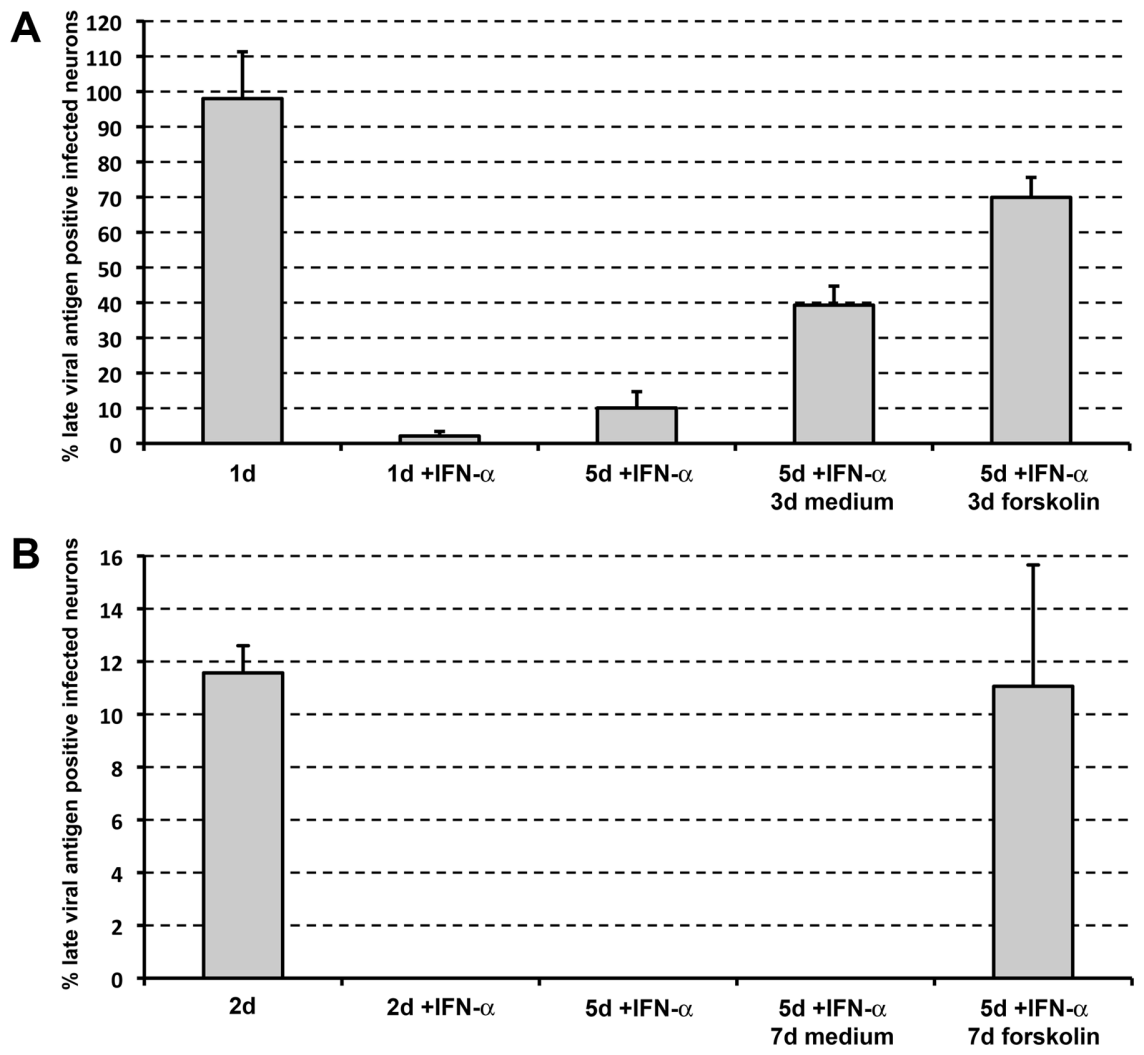
IFNalpha induces a reactivatable, latent PRV and HSV-1 infection in porcine TG neurons. Percentage infected neurons that are late viral antigen positive at 1, 5 and 8dpi with PRV (A) and at 2, 5 and 12dpi with HSV-1 (B) in the presence or absence of 500 U/ml IFNalpha. For the neurons fixed at 8dpi with PRV and 12dpi with HSV-1, medium containing IFNalpha was washed out at 5dpi and replaced with new culture medium or new culture medium supplemented with forskolin (200 µM). Data represent the mean ± s.e.m. of three independent experiments.

The effect of IFNalpha on the expression of late viral proteins upon inoculation with both viruses was analyzed. Two-chamber systems were pretreated with IFNalpha for 24 h and after infection, IFNalpha remained present in the inner chamber for the entire experiment. A dose dependent decrease was observed in the number of PRV infected neurons expressing late viral proteins at 24hpi, ranging from 36±1% late viral antigen positive neurons with 0.5 U/ml IFNalpha (data not shown) to 2±1% with 500 U/ml (Fig. 2A). The latter concentration was selected for all further experiments. The suppressive effect of IFNalpha on PRV replication was sustained over a longer period of time since at 5dpi, still only 10±4% of infected neurons were late viral antigen positive (Fig. 2A). For HSV-1, the suppressive effect of IFNalpha was even more pronounced since not a single HSV-1 gD positive infected neuron was detected, both at 2 and 5dpi (Fig. 2B). Both for PRV and HSV-1, in the absence of IFNalpha, cytopathic effect was complete well before 5dpi. These results indicate that IFNalpha is able to efficiently suppress alphaherpesvirus replication in porcine TG neurons for several days.

### Interferon alpha suppresses PRV IE180 and HSV-1 ICP4 protein expression in TG neurons

To determine whether IFNalpha not only suppresses late viral protein expression, but may also affect IE protein expression, levels of ICP4 of HSV-1 or the corresponding IE180 protein of PRV were analyzed in IFNalpha–treated two-chamber systems. For HSV-1, IFNalpha treatment resulted in fast and efficient suppression of ICP4 protein levels, leading to undetectable ICP4 levels at both 2dpi and 5dpi. For PRV, IFNalpha-mediated suppression of IE180 appeared to be less efficient and slower, resulting in 91.5±14.8% of IE180-positive neurons with axonal outgrowth in the outer chamber at 1dpi, and 46.1±3.2% of IE180-positive neurons at 5dpi. These results indicate that IFNalpha is able to suppress alphaherpesvirus IE protein levels in porcine TG neurons, and that this appears to occur more efficiently in HSV-1-infected neurons compared to PRV-infected neurons.

### Interferon alpha induces a stably suppressed quiescent PRV and HSV-1 infection

During virus latency, the virus is present in a stably suppressed state. In other words, latency persists, even when the suppressive agent is removed. To analyze whether IFNalpha is able to induce such a stably suppressed state of alphaherpesvirus infection, we analyzed whether or not withdrawal of IFNalpha at 5dpi resulted in re-expression of late viral antigens in TG neurons. For PRV, infection was stably suppressed in 60% of the neurons, as they did not express detectable levels of late viral proteins at 3 days post IFNalpha withdrawal (Fig. 2A). For HSV-1, none of the neurons initiated expression of detectable levels of HSV-1 gD even at 7 days after withdrawal of IFNalpha (12dpi) (Fig. 2B), showing that all HSV-1 infected TG neurons were in a stably suppressed quiescent state of infection.

### Forskolin treatment triggers PRV and HSV-1 reactivation in neurons containing quiescent virus

Alphaherpesvirus latency is defined as a functional viral genome retained in neurons in the absence of virus particles but capable to reactivate resulting in production of new infectious virus [4]. Forskolin is a known stimulus of alphaherpesvirus reactivation [19]–[21]. Therefore, we analyzed whether forskolin was able to reactivate PRV and HSV-1 in the neurons containing stably suppressed virus. Again, IFNalpha was withdrawn at 5dpi and medium supplemented with forskolin (200 µM) was added. For PRV, 70% of the infected neurons were late viral antigen positive at 3 days post IFNalpha withdrawal (Fig. 2A), often with virus spread to neighbouring non-neuronal cells, indicating that forskolin treatment induced reactivation of PRV in 50% of neurons that contained stably suppressed virus at 5dpi. For HSV-1, a similar experiment was performed but medium supplemented with forskolin was added twice, at 5dpi (when IFNalpha was withdrawn) and again at 8dpi. Analysis of neurons at 12dpi showed that forskolin had induced reactivation of HSV-1 since 11% of infected neurons were positive for HSV-1 gD at that time point (compared to 0% without forskolin) (Fig. 2B), often with spread of the virus to neighbouring non-neuronal cells. Reactivation ultimately led to complete cytopathic effect in the inner chamber (data not shown). Forskolin triggers reactivation but was found not to prevent the IFNalpha–mediated suppression of productive viral replication, since addition of IFNalpha and forskolin in parallel did not influence the ability of IFNalpha to suppress productive viral replication (data not shown). Overall, these data show that forskolin can reactivate HSV-1 and PRV from IFNalpha–induced quiescence.

### PRV and HSV-1 express LATs during *in vitro* latency-like quiescence

**Figure 3:**
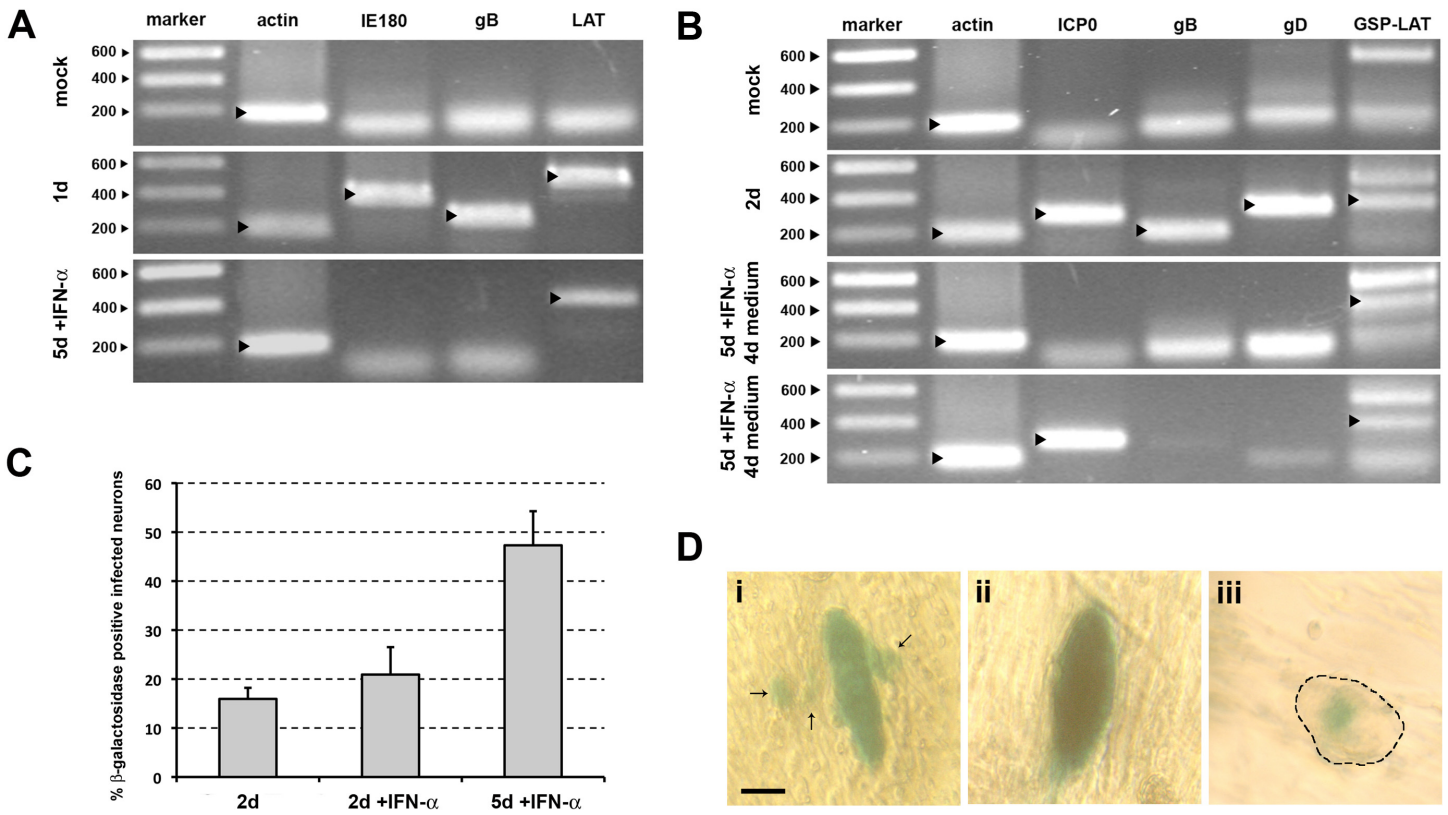
PRV and HSV-1 express LATs during *in vitro* latency. (A,B) RT-PCR analysis of actin and viral immediate early (IE180 and ICP0), late (gB and gD) and LAT transcript RNA isolated from neuronal cultures that were either mock infected, productively infected with PRV (A, 1dpi) or HSV-1 (B, 2dpi), or latently infected with PRV (A, 5dpi with IFNalpha) or HSV-1 (B, 9dpi, 4 days post IFNalpha withdrawal). For each condition three different samples were analyzed and representative gels are shown. For HSV-1, two samples of 9dpi, 4 days post IFNalpha withdrawal are shown, one without and one with detectable ICP0 transcript expression. Specific bands are marked with a black arrowhead. (C) Percentage of infected neurons positive for LAT promoter-driven beta-galactosidase at 2 and 5dpi with HSV-1 LbetaA in the presence or absence of 500 U/ml IFNalpha. Data represent the mean ± s.e.m. of three independent experiments. (D) Light microscopic images of uniform (i,ii) and focal (iii) LAT promoter-driven beta-galactosidase distribution during the acute stage (2dpi without IFNalpha, i, ii) or the latent stage (5dpi with IFNalpha, iii) of infection with HSV-1 LbetaA. Arrows point to infected non-neuronal cells (i), dashed line marks contour of neuronal cell body in (iii) (bar  = 20 µm).

During HSV-1 and PRV latency *in vivo*, expression of LATs is frequently observed [22], [23]. To determine LAT expression during *in vitro* latency-like quiescence by wild type HSV-1 and PRV, RT-PCR was performed on RNA isolated from TG neurons derived from these two-chamber models that were i) mock infected, ii) productively infected, and iii) uniformly quiescently infected (Fig. 3A,B). For PRV, all three quiescently infected cultures (CPE negative at 5dpi) examined were negative for immediate early (IE180) and late (gB) viral RNA but one was positive for LAT RNA (Fig. 3A). For HSV-1, all three quiescently infected cultures examined were negative for late (gB and gD) viral RNA but 2 were positive for LATs (Fig. 3B). RT-PCR assays contained quite prominent aspecific amplification signals, which are likely due to the high number of PCR cycles needed to detect sufficient signal. Specificity of the bands was confirmed via sequencing and contamination by viral genomic DNA was excluded by DNAseI treatment and by performing control reactions in the absence of reverse transcriptase. The RT-PCR assays show that both PRV and HSV-1 express detectable levels of LATs in a subset of infected porcine TG neurons during IFNalpha induced latency-like quiescence. Interestingly, one of the three quiescently HSV-1 infected cultures examined, which were all negative for gD RNA, was positive for ICP0 transcripts (Fig. 3B).

### The HSV-1 LAT promoter is activated over time during latency-like *in vitro* quiescence

For HSV-1, LAT promoter activity was assessed during IFNalpha induced latency-like quiescence *in vitro*. Two-chamber systems were infected with the LbetaA HSV-1 mutant carrying a LacZ reporter gene under control of the LAT promoter [16] in the presence or absence of IFNalpha. X-gal staining of cultures not treated with IFNalpha showed that 16% of infected neurons were beta-galactosidase positive at 48hpi (Fig. 3C). This percentage is similar to the percentage of HSV-1 gD positive neurons after wild type HSV-1 infection at 48hpi (12%). In the presence of IFNalpha, we observed an increase of beta-galactosidase positive infected neurons over time to about 50% positive neurons at 5dpi (Fig. 3C). As a control, no evidence for beta-galactosidase activity was found at 5dpi in the presence of IFNalpha in neurons infected with the SΔUS5-LacZ HSV-1 mutant carrying the reporter gene LacZ under control of the human CMV MIEP promoter. The activation of the LAT promoter over time suggests a gradual de-repression of the LAT promoter. Analysis of beta-galactosidase distribution in ±20 neurons in either lytically infected neurons (2dpi in the absence of IFNalpha) or quiescently infected neurons (5dpi in the presence of IFNalpha) indicated that in lytically infected neurons, beta-galactosidase distribution was generally uniform throughout the neuronal cell body (Fig. 3Di, ii) and sometimes associated with spread of infection to surrounding non-neuronal cells (Fig. 3Di), while a more focal beta-galactosidase distribution was frequently found in quiescently infected neurons (Fig. 3Diii). In summary, in the presence of IFNalpha, HSV-1 LAT promotor activity can be observed in up to ±50% of infected neurons at 5dpi. Since this percentage is higher than the percentage of neurons that proceed to a productive replication in the absence of interferon (12%, Fig. 1F), this suggests that at least a fraction of the neurons that do not show the capacity to proceed to productive replication in the absence of IFNalpha do show LAT promoter activity during prolonged incubation with IFNalpha.

## Discussion

In the current report, we show that addition of IFNalpha to two-chamber systems of porcine TG neurons is sufficient to induce a quiescent PRV and HSV-1 infection that shows strong similarities to *in vivo* latency.

Besides the insights in alphaherpevirus latency/reactivation generated by in vivo studies in animals, ex vivo explants of latently infected neurons, and from biopsies of deceased individuals, over the years, several very valuable in vitro models have been developed that have increased our understanding of HSV quiescence and latency. Some of the most notable of these in vitro systems are based on the use of antiviral drugs, mainly nucleoside analogues that act as viral DNA chain terminators. Such drugs have been shown to induce a stably suppressed state of HSV infection that shows similarities to *in vivo* latency. Cells used in these models vary from fibroblasts over neuronal cell lines (e.g. PC12) to primary rat dorsal root ganglion neurons [24]–[28]. Like noncytotoxic lytic granules and type II interferon, antiviral drugs like acyclovir have also been shown to have the capacity to prevent HSV reactivation in vitro upon explantation of TG neurons obtained from latently infected mice [29]–[31]. Some of the other *in vitro* models of HSV latency are based on the use of replication-defective, attenuated virus strains [32]. Still other models are based on a reversible, temperature-dependent suppression of virus replication [33], [34]. Some previous in vitro latency models contained IFN – however, simultaneous addition of nucleoside analogues was required to establish quiescence [25]–[27]. Since addition of nucleoside analogues without IFN also prevents viral replication and leads to quiescence [28], these data were inconclusive on a potential involvement of IFN in latency establishment. In our current model, addition of IFNalpha is sufficient to establish latency-like quiescence, without the need for nucleoside analogues. This important difference is perhaps due to the use of a two-chamber system that allows an in vivo-like route of infection of neurons, via retrograde axonal spread. It has been suggested before that the long distance retrograde transport of HSV in neurons results in reduced levels of the viral VP16 transactivator reaching the nucleus [35]. Without a two-chamber system, virus can access the neurons via the cell body, thereby circumventing retrograde axonal spread.

The obtained results indicate a strong similarity between the currently described *in vitro* quiescence and alphaherpesvirus latency *in vivo*. First of all, our results show that both PRV and HSV-1 express detectable levels of LATs in a subset of quiescently infected *in vitro* systems. It will be interesting to further explore LAT expression in the *in vitro* latency model, e.g. by determining nuclear localization of the LAT transcripts. Our observation that not all cultures contain detectable LAT expression appears to be in line with the notion that *in vivo* a varying number of latently HSV-1 infected human TG neurons expresses detectable levels of LATs [36]. We observed that some quiescently infected cultures are positive for both LAT and ICP0 transcripts. Although we cannot formally rule out the possibility that these LAT/ICP0 mRNA double-positive quiescent cultures may consist of neurons that express either LAT or ICP0 mRNA, it is also possible that quiescently infected neurons express both transcripts at the same time. Interestingly, there is increasing evidence for the presence of ICP0 transcripts in ganglia of humans and mice latently infected with HSV-1 [37]–[39], and our current data may therefore be in line with indications that ICP0 protein expression in latently infected neurons may at least partly be blocked at the post-transcriptional level, probably due to microRNA activity of LATs and perhaps other, unknown, viral and host factors [37], [39]–[41]. Using a previously described HSV-1 recombinant expressing beta-galactosidase under control of the LAT promoter [16], a focal beta-galactosidase distribution was observed in latently infected neurons. Such a focal beta-galactosidase distribution was also observed during *in vivo* latency in ganglia of mice latently infected with the LbetaA mutant and with another HSV mutant expressing LacZ under control of the LAT promoter [16], [42]. It has been suggested that the focal beta-galactosidase distribution may be due to physiological differences between lytically and latently infected neurons.

Our results indicate that IFNalpha leads to efficient establishment of PRV and HSV-1 latency-like quiescence in TG neurons *in vitro*. Based on these data, it is tempting to speculate that IFNalpha also represents a key immune component involved in the efficient establishment of alphaherpesvirus latency in sensory neurons *in vivo*. Some in vivo data may support this hypothesis: several reports indicate that impaired IFN responses *in vivo* can result in uncontrolled lytic virus replication and, often fatal, afflictions of the central nervous system, including herpes simplex encephalitis in humans [43], [44], and increased replication efficiency of strongly attenuated HSV-1 mutants in TG neurons of mice [45]. Based on the current in vitro data, it will be interesting to further investigate if type I IFNs indeed act as a double-edged sword in vivo: on the one hand protecting the host from severe infection by alphaherpesviruses, on the other hand contributing to their lifelong persistence in a latent form.

*In vivo*, there is evidence that type II IFN (IFNgamma), mainly produced by CD8^+^ T lymphocytes, plays only a minor additional role to type I IFNs in controlling early acute infection [45], [46] but is important to prevent reactivation of alphaherpesviruses from latency [31], [47]. Further in line with the similarity between alphaherpesvirus *in vivo* latency-like quiescence and the currently described *in vitro* model, we found that IFNgamma, although unable to suppress PRV replication over longer periods of time and therefore unable to induce PRV quiescence *in vitro* as observed for IFNalpha, is able to maintain PRV quiescence *in vitro* (data not shown). Although speculative at this point, together with the literature data, this may suggest that especially type I IFNs, as a crucial factor of the innate antiviral immunity, may be of importance during establishment of latency whereas type II IFN produced by the adaptive immunity are of crucial importance in maintaining the virus in a latent state and preventing reactivation. It will be interesting to determine whether the latency-like quiescent state of infection is the result of IFNalpha directly promoting the establishment of latency/quiescence, or, alternatively, of an IFNalpha-mediated inhibition of lytic replication that indirectly promotes latency/quiescence. Dissecting which IFNalpha-induced effectors are involved in the induction of quiescence will aid to clarify this. Both in PRV and HSV, IFNalpha suppressed protein expression levels of IE genes ICP4/IE180, suggesting that IFNalpha–mediated IE suppression may be a key element in the ability of this innate immune effector to establish quiescence. In line with this hypothesis, we observed that the percentage of neurons that still show detectable IE180 protein levels at 5dpi with PRV in the presence of IFNalpha correlates well with the percentage of neurons that are not in a stable quiescent infection at that time point and proceed to productive replication upon IFNalpha withdrawal (46% versus 40%).

In summary, this study presents a novel and unique *in vitro* system to dissect aspects of the latency/reactivation cycle of wild type alphaherpesviruses and points to IFNalpha as a potential driving force in efficient alphaherpesvirus latency establishement. In addition, the currently described in vitro model may provide a unique tool to screen possible drug candidates that interfere with the latency/reactivation cycle.
